# De-novo fabrication of sunlight irradiated silver nanoparticles and their efficacy against *E. coli* and *S. epidermidis*

**DOI:** 10.1038/s41598-021-04674-x

**Published:** 2022-01-13

**Authors:** Hammad Arshad, Saima Sadaf, Umer Hassan

**Affiliations:** 1 Department of Electrical and Computer Engineering, School of Engineering, Rutgers, The State University of New Jersey, Piscataway, NJ USA; 2 School of Biochemistry and Biotechnology, University of the Punjab, 54590 Lahore, Pakistan; 3 Global Health Institute, Rutgers, The State University of New Jersey, New Brunswick, NJ USA; 4 Department of Biology, Lahore Garrison University, Lahore, Pakistan

**Keywords:** Biotechnology, Engineering

## Abstract

Silver nanoparticles (AgNPs) gained significant attention due to their activity against microbial pathogens, cancer cells, and viral particles etc. Traditional fabrication methods require hazardous chemicals as reducing agents and their usage and disposal pose a significant hazard to environmental ecosystem. Here, a de novo, robust, cost effective and an eco-friendly method is reported to fabricate AgNPs irradiated with sunlight (SL) while using *Salvadora persica* root extract (SPE) as reducing agent. Sunlight (SL) irradiated *S. persica* silver nanoparticles (SpNPs) i.e., SL-SpNPs were characterized using multiple techniques and their antibacterial efficacy was evaluated. The SL-SpNPs were synthesized in 10 min. Field emission scanning electron microscopy (FE-SEM) and transmission electron microscopy (TEM) analysis revealed their spherical morphology with a size range of 4.5–39.7 nm, while surface plasmon resonance (SPR) peaked at 425 nm. Fourier transform infrared spectroscopy (FTIR) analysis suggested that the reduction of SL-SpNPs was due to the presence of phytochemicals in the SPE. Furthermore, X-ray powder diffraction (P-XRD) pattern depicted the crystal structure of SL-SpNPs, hence proving the presence of AgNPs. Further the antibacterial studies were carried out against *Escherichia coli* (ATCC 11229) and *Staphylococcus epidermidis* (ATCC 12228) using Kirby Bauer method. The minimum inhibitory concentration (MIC) and minimum bactericidal concentration (MBC) for *E. coli* were determined to be 1.5 μg/mL and 3.0 μg/mL respectively while MIC and MBC values for *S. epidermidis* were found to be 12.5 μg/mL and 25 μg/mL respectively. The solar irradiation-based fabrication method and resulting SL-SpNPs can find their utility in many biomedical and environmental applications.

## Introduction

Environment friendly and robust methods for nanomaterial fabrication with variety of applications has given a revolutionary uplift to the field of nanobiotechnology^1–3^. Among many metallic nanoparticles, silver nanoparticles (AgNPs) have gained a significant reputation owing to their broad-spectrum biomedical, environmental, and antimicrobial applications^4–7^. More specifically, antimicrobial applications of AgNPs against multi drug resistant (MDR) pathogens has gained a remarkable attention recently. The synthesis of active AgNPs against MDR pathogens with lesser cytotoxicity on mammalian cells is of tremendous significance in therapeutic applications^8,9^. Biological (i.e., microbial, plant based etc.) fabrication of nanoparticles (NPs) has gained superiority over chemical or physical methods because of being non-hazardous, eco-friendly, faster, and simpler methods. Further, plant-based synthesis is advantageous over microorganisms mediated synthesis due to being non-toxic and ease of production^1,10^. Medicinal plants contain many phytochemicals such as phenolics, flavonoids, terpenes etc. which may enhance the biological activity of metallic NPs in addition to their utility in reduction and stabilization of NPs. The cytotoxicity of such plants is considerably low and considered as safe for humans when used for NPs synthesis^1,2^. However, there are some limitations of metallic NPs when used for biomedical applications. The main disadvantage is the genotoxicity of the NPs in time and dose dependent manner^11^. The production of ROS is likely the main reason of cell death and in some studies, radicalization by NPs leads to redox reactions resulting in programmed cell death^12^. Therefore, in vivo studies are critical to determine the application of NPs and the synthesis approach will likely define their utility and efficacy in such applications.

*Salvadora persica* (*S. persica*), a plant found in South Africa and Asian countries^13^ has been used in this study for fabrication of AgNPs. Traditionally, the plant is locally known to have applications such as sedative effects, antiulcer, anticonvulsant but most commonly for the dental hygiene^14,15^. In customary areas, the dental cleaning with roots and twigs of *S. persica* has also been recommended by World Health Organization^16^ in specific circumstances. Aqueous extracts of roots, leaves and bark has been used in studies as a single component for reducing and capping of AgNPs without the need of any other chemicals^14,17–19^. Recently, synthesis of AgNPs against microbial pathogens by using of *S. persica* roots extract with aqueous AgNO_3_ was reported^20^ while employing high temperature heating for the nanoparticle synthesis.

Many variations in the process for synthesis of metallic NPs have been reported such as agitation, heating, microwave assisted, and autoclave mediated etc^20–22^. In contrast, a routine source of energy, i.e., sunlight has also been explored as an inducive source for the synthesis of metallic NPs (silver, gold, nickel etc.)^23–25^. The solar irradiation mediated synthesis has been robust, cost effective and results in efficient AgNPs production^26,27^. The main advantage of using solar light irradiation for AgNPs synthesis is a resulting non-toxic, eco-friendly chemical process^24,28^.

In this work, sunlight irradiation was employed for robust synthesis of AgNPs using SPE under ambient conditions. The roots of *S. persica* have been used to fabricate AgNPs with heating methods which are time consuming (few hours)^19,20^. Here, a robust method of sunlight irradiated AgNPs synthesis has been reported using *S. persica* extract as reducing and capping agent. The study also presents a detailed antimicrobial assay on two bacterial pathogens (gram positive and gram negative) using disc diffusion method and determined their minimum inhibitory concentration (MIC) and minimum bactericidal concentration (MBC), which were lacking in many of the previous studies (comparative analysis is provided in Table 1). The AgNPs produced with the current method carry significant antibacterial potential which in future may be employed for biomedical and environmental applications. Moreover, the methodology may provide new horizons in the disposal of NPs for applications where leaching of NPs is a challenge to address, for example, immobilized AgNPs in the polymers employed for water treatment.

**Table 1:** Comparison of sunlight mediated AgNPs using plant extracts.

References	AgNO_3_ Conc. (mM)	Plant extract and concentration (%; w/v)	Incubation temp. ℃/time	Shape	Size (nm)	Antimicrobial studies			
						Bacterial strain	NPs for ZOI (µg/ml, ml etc.)	ZOI (mm)	MIC (µg/ml)
^39^	01	*Ocimum sanctum Linn*/7	28 /60 min	Spherical	7–11	*S. aureus*	NA	NA	NA
^38^	03	*Sida retusa/10*	35/30 min	Spherical	20–70	*E. coli, S. typhi, B. subtilis and S. aureus*	12 μg/ml	15, 15, 14 and 17	NA
^27^	01	*Polygonatum graminifolium*/2	37/30 min	Spherical, triangular	3–15	*S. aureus, E. coli*	80 µl	16, 27	NA
^29^	01	*Allium ampeloprasum/*1	18–20/20 min	Spherical, triangular	35	*Candida albicans (5 strains)*	2.5–10 μg/ml	15–20	32.6
^57^	01	*Rivina humilis*/10	-	Spherical (clustered)	51	*S. aureus, B. cereus and B. subtilis, E. aerogenes, E. coli and S. flexneri*	800 µg/ml	8.5, 11.7, 8 10.8, 11, 10.5	NA
^58^	5	*Tilia cordata, Matricaria chamomilla, Calendula officinalis and Lavandula angustifolia*/10	-	Spherical	50	*S. aureus, E. coli*	NA	11 ± 0.0, 10 ± 0.3, 12 ± 0.5, 15 ± 0.1, 16 ± 0.0	NA
^59^	0.75	*Jasminum subtriplinerve Blume/5*	RT/150 min	Spherical	20–50	*B. cereus, E. coli, P. aeruginosa, S. aureus, and* *Salmonella*	NA	13.0, 14.2, 19.5, 10.0 & 15.3	33.1, 8.3, 8.3, 33.1 & 8.3
^34^	100	*Allium sativum/10*	15 min	Spherical	3.02–10.89	*B. cereus, P. aeruginosa, P. chrysogenum, A. niger, and A. flavus*	100—6.25 µg/ml	18, 25, 12.67 ,14.33 &13.0	NA
Current Study	10	*Salvadora persica/*10	RT/10 min	Spherical	4.5–39.7	*E. coli & S. epidermidis*	20 µg	11.5 ± 0.5 & 18.5 ± 0.5	1.5 & 12.5

## Methods

### Materials, chemicals, and microorganisms

Roots of *S. persica* and sterile syringe filters (0.45 µm pore size) were acquired from Amazon, ASIN: B00IN6RSH6 and ASIN: B06Y15LBZ5 respectively. Silver nitrate (99.9% pure) was supplied by Sigma Aldrich, USA (209139-25G). Pure pallets of gram-negative *Escherichia coli* (ATCC 11229) and gram-positive *Staphylococcus epidermidis* (ATCC 12228) were purchased from Microbiologics^®^, catalog no. 0681 (Kit contains: 5 pellets of a single enumerated strain and 5 vials of hydrating fluid (1.2 ml per vial) and 0371P (2 self-contained units of a single organism), respectively. Blank and Chloramphenicol discs of 6 mm were purchased from Hardy Diagnostics^®^, Z7121 and Z8341 respectively while nutrient agar plates were supplied by Evviva Sciences™ (ASIN: B07CSKBD8T). The use of plant materials (Roots of *S. persica*), their procurement and processing are in accordance with the institutional guidelines. Upon completion of experimental work, nanoparticles synthesized using plant materials and left-over roots of *S. persica* were disposed according to biosafety guidelines established by the university.

### Synthesis of sunlight irradiated silver nanoparticles (SL-SpNPs)

Thin sections of *S. persica* roots were finely cut with a sharp blade and thoroughly washed with deionized (DI) water followed by overnight drying at 50 ℃ in an oven. A 10 g of dried *S. persica* sections were taken in 100 ml of deionized (DI) water and subsequently boiled for 15 min with repetitive mixing by using a magnetic stirrer. The resulting *S. persica* extract (SPE) was brought to room temperature (RT) followed by filtration with a 0.45 µm syringe filter (sterile) and placed in a sterile container at 4 ℃ until further use.

The synthesis of NPs was studied using sterile 24 well polystyrene plates. A 100 mM solution of silver nitrate (SN) was prepared by mixing 16.9 g of silver nitrate (99.9%) in 1000 ml of DI water and stored in amber bottle in a fume hood. SN was further diluted to make 1 mM, 2 mM, 3 mM, 4 mM, 5 mM, 10 mM, 20 mM and 50 mM solutions and stored in dark. These concentrations of SN were mixed with SPE in a fixed ratio of 9 to 1. The reaction mixture was exposed to sunlight inside the lab at RT (25 ℃). Similar irradiation methods have been reported earlier as well^29^. The change in color was noted from 0 to 60 min and reaction was stopped by placing the plate in a dark. Considering 10 mM as a mid-range and optimized condition for the NPs production;^20^ the time based reduction of silver ions to AgNPs from 1 to 10 min was monitored. Further, NPs were centrifuged and subsequently washed with DI water three times to remove any leftover impurities.

### Characterization

Initially fabricated nanoparticles i.e., SL-SpNPs were analyzed using a single beam spectrophotometer (VWR UV-1600) equipped with a multi-wavelength professional software package for UV–Vis spectroscopy within range of 300–800 nm. Dynamic light scattering (DLS) analysis was carried using plastic disposable cuvettes for size distribution study of the NPs by utilizing Malvern Panalytical’s Zetasizer Ultra. An Agilent Handheld Spectrometer (4300) was used for Fourier transform infrared spectroscopy (FTIR). A 50 µl of each aqueous sample (NPs and SPE) was positioned at the diamond ATR (attenuated total reflectance) assembly and evaluated. Crystallographic structure was studied by X-ray powder diffraction (P-XRD) and was performed by operating Bruker Vantec-500 area detector and a Bruker FR571 rotating-anode X-ray generator operating at 40 kV and 45 mA and equipped with a 3-circle Azlan goniometer (Cu Ka; l = 1.5418 Å) at 25 °C (RT). Data was collected with a sample-to-detector distance of 10 cm. The scan range was varied from 20 to 80 with ω scan of 2°. The morphological analysis was evaluated by field emission scanning electron microscopy (FE-SEM) using Zeiss Sigma Field Emission SEM with Oxford INCA Penta FETx3 EDS system, (Model 8100) with fully digital image collection, transfer and analysis. Briefly, the sample was diluted at a concentration of 0.1 µg/µl in DI water followed by pipetting a drop on a silicon wafer (10 mm) attached with aluminum stubs (16111, Ted Pella, Inc.) with carbon tape (16084-1 PELCO Tabs™) adhered. Voltage level of 3 kV was used to analyze the samples. AMT-XR11 digital camera with Philips CM12 was used for transmission electron microscopy (TEM) characterization where 80 kV acceleration was used to collect images. A drop of 10 µg/ml concentration of NPs was applied on copper grid holding carbon film (VWR, 100503-154) and kept in a desiccator overnight at RT.

### Antibacterial assay

*Escherichia coli * (ATCC 11229) and *S. epidermidis* (ATCC 12228) were used to evaluate antibacterial efficacy of the fabricated nanoparticles. Nutrient agar was used to culture the bacteria in all experiments following standard protocol^30^. The antimicrobial assay was based on Kirby Bauer susceptibility method^31^. The initial inoculum was prepared (as the optical density at 600 nm (OD_600_) value equals 0.1 which is indicative of ~ 1 × 10^7^ CFU/ml concentration) for both strains in sterile PBS (phosphate buffer saline) followed by homogenous spreading utilizing sterile cotton swabs on the agar plates. Synthesized NPs (1–5 mM, 10 mM, 20 mM and 50 mM) were applied on both bacterial pathogens with an equal volume of NPs solution i.e., 20 µl and incubated overnight at 37 °C. Zones of inhibition were subsequently measured in mm using scale. The similar method was employed for selected NPs in comparison with SN (10 mM) and SPE where 20 µg/ml of NPs and 20 µl for each SN and SPE was used. Chloramphenicol (C) 30 µg disc was used as positive control in all experiments.

Broth assay was employed for MIC and MBC studies. NPs were serially diluted to make a concentration of 0.19–25 µg/ml in nutrient broth in a 24 well plate of polystyrene. Freshly cultured bacteria were suspended in sterile PBS to get a concentration of ~ 1 × 10^7^ CFU/ml. Two µl of that suspension was inoculated in each well except blanks. Bacteria without nanoparticles were used as negative controls. The mixture was incubated overnight at 37 °C followed by CFU count on agar plates and OD_600_ measurements. The minimum quantity of NPs which resulted in 98% growth inhibition was termed as MIC, whereas the next higher NPs concentration was considered as MBC since it resulted in a complete bacterial growth reduction. Experiments were conducted in triplicates in aseptic conditions.

## Results

### Synthesis

**Figure 1:**
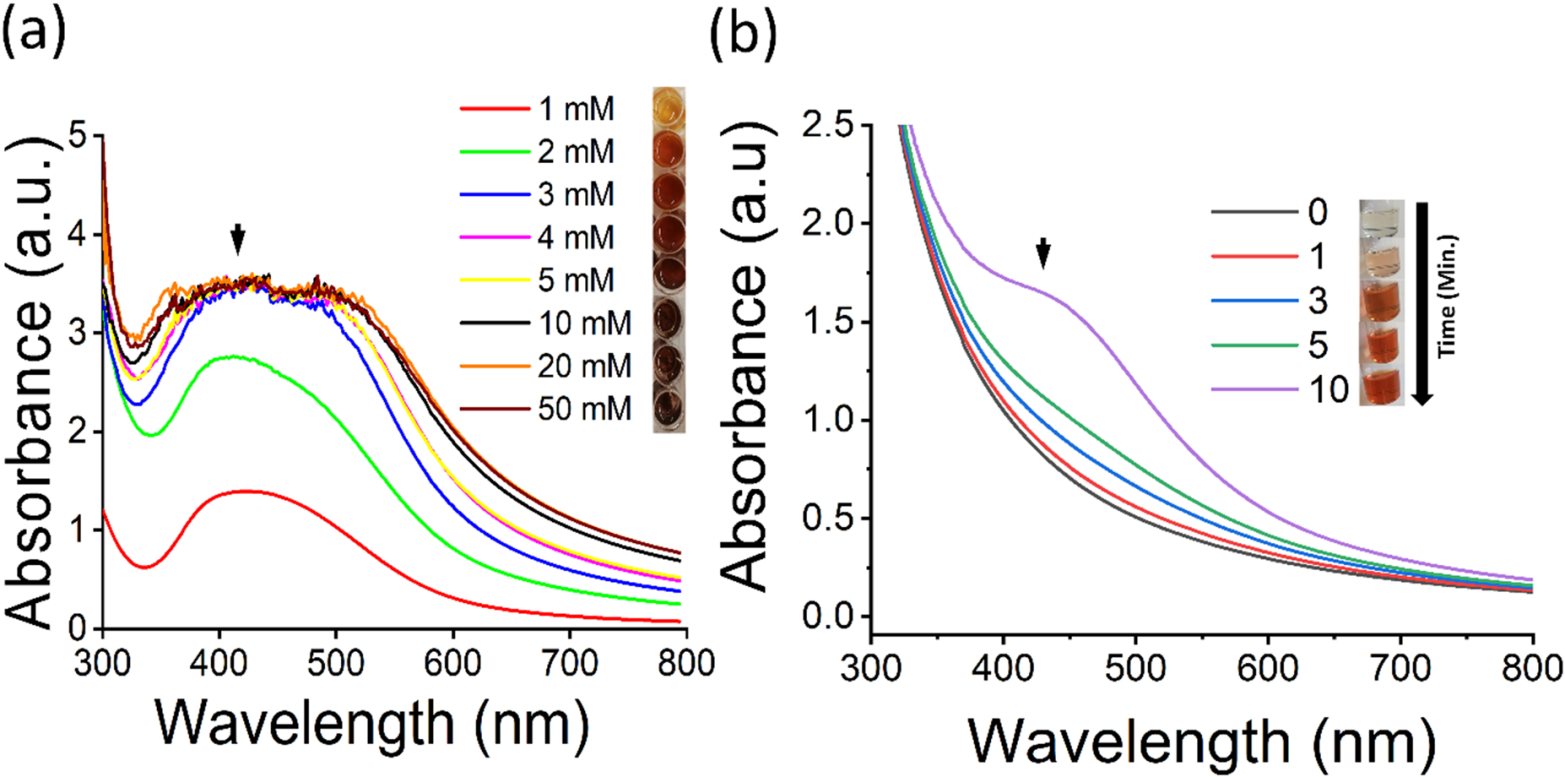
UV–Vis spectrum analysis. (**a**) UV–Vis spectrum of SL-SpNPs using various SN molarities (1–5, 10, 20, 50) mM with sunlight irradiation for 1 h. All samples show absorbance peaks between 400 and 450 nm as shown by arrow. (**b**) The synthesis of SL-SpNPs (with 10 mM SN) with varying times of incubation. UV–Vis analysis shows the highest value after 10 min of incubation (arrow).

Nanoparticles were synthesized using various silver nitrate (SN) molarities (1–5 mM, 10 mM, 20 mM, 50 mM) and 10% (w/v) SPE from 1 to 60 min under sunlight at RT without any stirring or mixing. The change of color was observed from yellow to brown in all samples after 60 min of incubation. This is shown by absorbance peak values from 400 to 450 nm as revealed from UV–Vis analysis in Fig. 1a. Further, UV–Vis spectrum for synthesis of SL-SpNPs (with 10 mM SN) with varying times of incubation is shown in Fig. 1b. SL-SpNPs were fabricated using 10 mM of SN and 10% (w/v) of SPE with a ratio of 9:1 at RT without stirring or heating in 10 min. UV–Vis. analysis shows the highest value of absorbance after 10 min of incubation as indicated by arrow in Fig. 1b.

### Characterization

**Figure 2:**
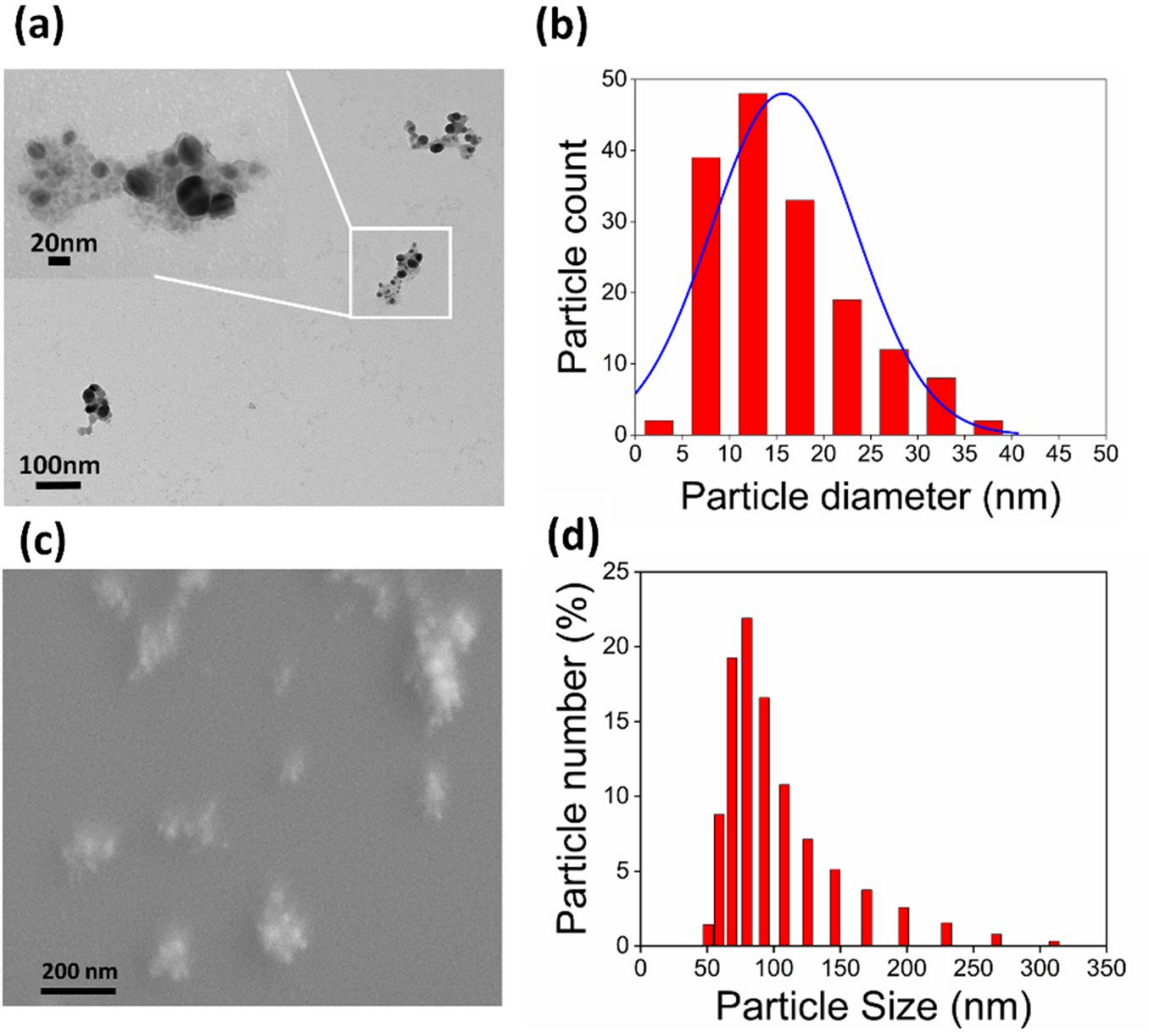
Size and morphology analysis. (**a**) TEM study depicts the spherical nature of the SL-SpNPs, inset shows the cluster of NPs stabilized by SPE. (**b**) The bar plot based on TEM analysis represents the size range of SL-SpNPs as 4.5–39.7 nm with an average of 15.38 nm. (**c**) Similarly, the FE-SEM analysis shows spherical nature of the NPs with small clusters. (**d**) However, the DLS analysis showed variable size percentage with an average of 79 nm of synthesized SL-SpNPs.

The morphological and size analysis by TEM is illustrated in Fig. 2a, b. A variable size in the range of 4.5–39.7 nm is observed for fabricated nanoparticles while depicting a spherical morphology. It is evident from Fig. 2a that SPE performed stabilizing effect on SL-SpNPs which holds the NPs together in small clusters. Similarly, FE-SEM image (Fig. 2c) represents the spherical nature of SL-SpNPs without any prominent aggregations. Moreover, DLS analysis confirmed the presence of variable sized SL-SpNPs in the suspension with a highest percentage of 79 nm NPs present (Fig. 2d).

**Figure 3:**
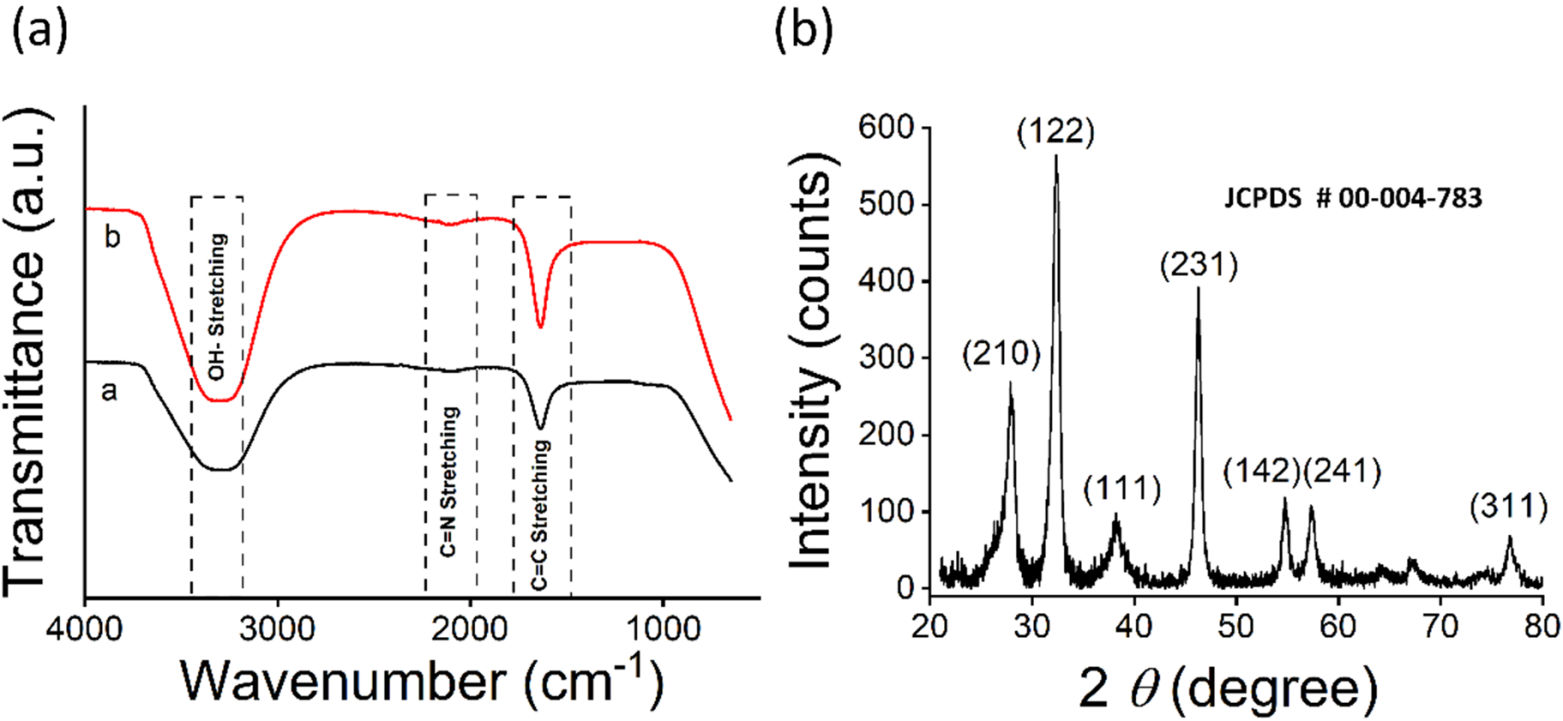
FTIR and XRD analysis. (**a**) The possible head groups of SPE phytochemicals involved in the bio reduction of SN are shown by FTIR analysis for SPE (black curve) and SL-SpNPs (red curve). (**b**) P-XRD results demonstrating the 2*θ* values confirming the lattice structure of SL-SpNPs.

The involvement of potential reducing phytochemicals was revealed by FTIR analysis (Fig. 3a) where bond stretching was observed at 3300 cm^−1^, 2100 cm^−1^ and 1630 cm^−1^ which corresponds to OH–, C=N– and C=C functional group heads. The enriched SPE may exhibit saponins, tannins, flavonoids etc. which are depicted by OH–. The presence of OH– containing reducing biomolecules is in agreement of previous studies on silver NPs production^16,32^. A similar pattern has been observed in case of C=N and C=C stretches which attributes to aromatic compounds containing nitrogen and carbonyl or carboxylic groups such as benzylamine, aniline etc. and others including amino acids and peptides^33,34^. The FTIR comparative analysis of SL-SpNPs (red transmission curve in Fig. 3a) and plant extract (black transmission curve in Fig. 3b) show presence of same bond stretching and functional groups e.g., OH–, C=C, thereby confirming the role of *S. persica* in fabrication (reduction and stabilization) of SL-SpNPs.

P-XRD analysis of SL-SpNPs shows characteristic peak values at 2*θ* = 27.86°, 32.34°, 38.2°, 46.2°, 54.74°, 57.3° and 76.72° which correlated to the lattice planes of (210) (122) (111) (231) (142) (241) and (311) as shown in Fig. 3b. The analysis confirmed lattice planes of the FCC crystal structure of synthesized SL-SpNPs according to the JCPD file number 00-004-783. Similar XRD patterns have been reported earlier as well^35,36^.

### Antibacterial assay

**Figure 4:**
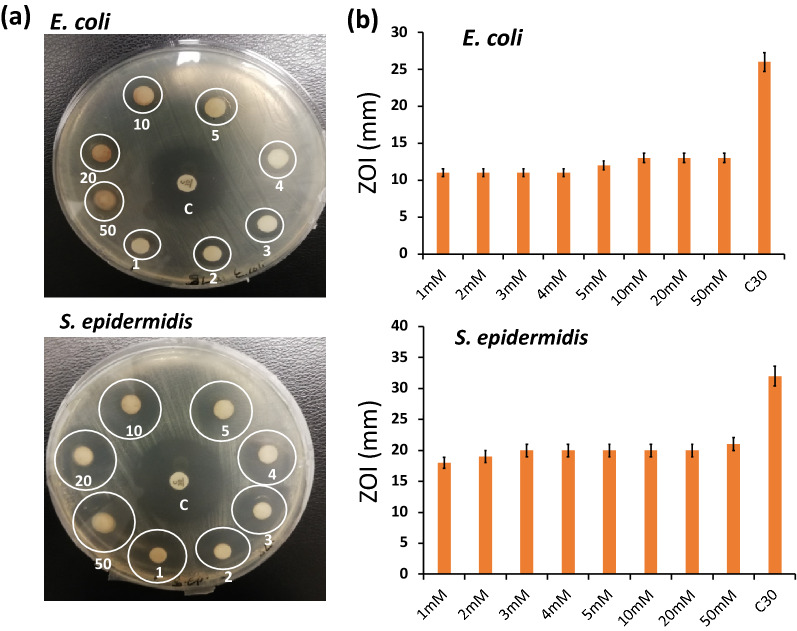
Antibacterial activity of SL-SpNPs on *E. coli* and *S. epidermidis*. (**a**) Antibacterial efficacy analysis was performed by using Kirby Bauer (disc diffusion) method. A 20 µl of eight (SL-SpNPs) samples were used i.e., 1 mM, 2 mM, 3 mM, 4 mM, 5 mM, 10 mM, 20 mM and 50 mM whereas antibiotic i.e., (chloramphenicol, **C** was used as a positive control in experiments. Zones of inhibition (ZOI) around the discs are evident for the effectiveness of SL-SpNPs against the bacterial pathogens. (**b**) ZOI were calculated by using a scale in mm and their mean values are shown by bar plots (error bars show standard deviation). Experiments were performed in triplicates.

The antibacterial activity of SL-SpNPs was quantified on both gram-positive and gram-negative bacterial pathogens. *E. coli* (gram-negative) and *S. epidermidis* (gram-positive) are commonly employed for this purpose. For antimicrobial study, Kirby Bauer’s disc diffusion method was employed to evaluate the efficacy of SL-SpNPs. The antimicrobial activity of the samples has been shown in Fig. 4 where zones of inhibition (ZOI) were noticed against all molarity samples (20 µl of samples were used). Antibiotic (chloramphenicol, **C**) was used as a positive control in all the experiments. Experiments were done in triplicates and their mean and standard deviation of ZOI were calculated. Figure 4b represents the bars which shows the mean values of ZOI, and error bars represents the standard deviations of ZOI.

**Figure 5:**
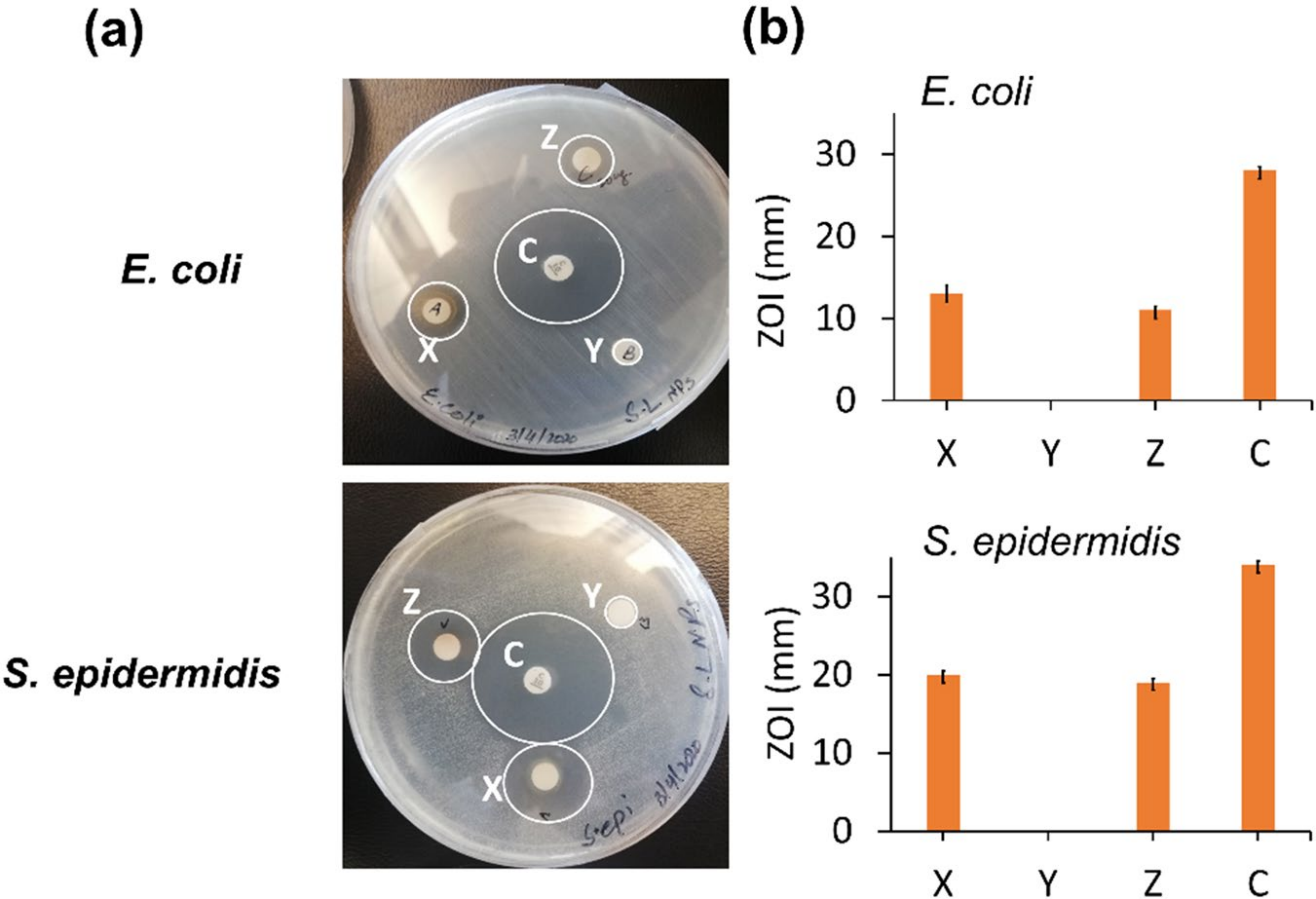
Antibacterial activity of silver nitrate solution, plant extract, AgNPs suspension, and chloramphenicol on *E. coli* and *S. epidermidis*. (**a**) Antibacterial efficacy analysis was performed by using Kirby Bauer (disc diffusion) method. Three samples were used i.e. (**X**) 20 μl of SN, (**Y**) 20 μl of SPE and (**Z**) 20 μg of SL-SpNPs whereas chloramphenicol, **C** was used as a positive control in all experiments. Zones of inhibition (ZOI) around the discs depicts microbial growth inhibition. (**b**) The ZOI were calculated by using a scale in mm and their mean value is shown by bar plots and standard deviation as error bars. No ZOI is found is found for sample Y.

In Fig. 5, disc diffusion method is used again to evaluate the antimicrobial efficacy using multiple samples including 20 μl of 10 mM SN (**X**), 20 μl of 10% (w/v) SPE (**Y**) and 20 μg of SL-SpNPs (**Z**) and positive control, chloramphenicol 30 μg (**C**). The ZOI noted for X, Y, Z and C were found to be 12 ± 1 mm, 0 ± 0 mm, 11.5 ± 0.5 mm, 28.5 ± 0.5 mm against *E. coli* and 19.5 ± 0.5 mm, 0 ± 0 mm, 18.5 ± 0 mm, 33.5 ± 0.5 mm against *S. epidermidis* respectively*.* The standard deviation and the mean values are represented as bar plots as shown in Fig. 5b. Further, no ZOI is observed for sample Y. The broth assay was used to evaluate MIC and MBC and the respective values were found to be 1.5 μg/ml and 3.0 μg/ml for *E. coli* whereas 12.5 μg/ml and 25.0 μg/ml for *S. epidermidis,* respectively.

## Discussion

The synthesis protocol was initially monitored by visual observation of color change in the sample mixtures of varying molarities of SN with constant SPE amount. The change into dark brown color was observed in all samples due to the bio-reduction of Ag^+^ to Ag^0^ which leads to the surface plasmon resonance (SPR) of synthesized NPs^19,21,37^. It was observed that all samples changed color within 1 h which has also been reported by other studies^38,39^. Further, it was observed that with increased SN molarity concentration, the color change is quicker. For example, sample containing 10 mM SN, the brown color change was observed only after 3 min of the incubation under sunlight. The change of yellow to brown color has been an indication of AgNPs production as reported in numerous studies because of the surface plasmon resonance of AgNPs^38,40,41^. Current study demonstrated that SPE can be used effectively to reduce SN into AgNPs with solar light irradiation without requiring other sources of energy such as mixing or heating, normally used^19,20,42^. Sunlight irradiation provided sufficient energy to fabricate silver nanoparticles at ambient conditions as also described by previous reports^38,39^. Nallal et al. reported ultraviolet radiation as one of the possible energy spectrum for nanoparticle synthesis^29^. The characteristic of AgNPs is to show a peak value between 400 and 450 nm when analyzed in a UV visible range^43^. SL-SpNPs depicted the similar behavior, where maximum excitation of AgNPs was observed at 425 nm as shown in Fig. 1b^6,44^. The increasing concentration of SN lead to the agglomeration of nanoparticles represented as noise in UV–Vis spectrum shown in Fig. 1b. Possible cause of this behavior may include excessive amount of Ag^+^ in the reaction mixture. The size and shape of SL-SpNPs are also comparable with the *Sida retusa* mediated AgNPs^38^ reported earlier. A detailed comparative analysis of our study with prior studies is shown in Table 1.

Earlier reports have also revealed the aggregation of the synthesized AgNPs^45–47^, however in the current study, FE-SEM analysis revealed SL-SpNPs to be spherical and polydisperse as shown in Fig. 2c. Similar behavior was noticed when actinobacteria was used for AgNPs synthesis under sunlight^48^. TEM analysis shows the presence of SPE around the NPs (Fig. 2b) that likely to keep SL-SpNPs in cluster form; thus, generating clusters of polydisperse NPs. The size distribution in TEM images was carried out using ImageJ software with total of 167 NPs. The bar plot diagram presents the presence of 4.5–39.7 nm with a mean of 15.38 nm (Fig. 2 b). Furthermore, the DLS analysis as shown in Fig. 2d shows the variation in size, with a mean size of approximately 79 nm. The variation in the size in DLS analysis may be attributed towards the presence of SL-SpNPs clusters in the suspension during analysis as compared to TEM analysis. FTIR analysis has informed the presence of major functional groups which are responsible in the reduction and stabilization of SL-SpNPs. The bond stretching revealed the involvement of amino acids, proteins, and peptides (C=C), reducing sugars (OH–) and other phytochemicals (CN–) present in SPE. The spectrum resemblance of plant extract (SPE) and SpNPs in Fig. 3a revealed the SPE involvement in the NPs reduction and stabilization. This is in agreement of similar studies reported earlier^19,42^.

The antimicrobial activity of SL-SpNPs (as demonstrated by disc diffusion studies shown in Figs. 4, 5) is significant against both bacterial pathogens (gram +ve and −ve) as compared to the previous studies^19^ as shown in Table 1. The presence of ZOI in all the samples confirmed that SL-SpNPs (1–5 mM, 10 mM, 20 mM and 50 mM) possess antibacterial activity (Fig. 4a) with slight increase in ZOI from lower to higher concentrations of SN (Fig. 4b). However, in a comparative analysis of SN, SPE and SL-SpNPs (10 mM) (Fig. 5a), it was noted that SPE resulted in no ZOI, while SN and SL-SpNPs showed significant antibacterial effect on the bacterial pathogens (Fig. 5b). The lack of antibacterial action of SPE was likely because of the lower concentration (10% w/v) used as compared to previous work^42^. Further, MIC and MBC values are considerably lower (2 orders of magnitude) as compared to Shaik et al.^19^ demonstrating higher antimicrobial potential and potential low toxicity. However, these values are greater than previous study using heat based synthesis of AgNPs^20^. The higher MIC and MBC as compared to previous study may be because of larger size of NPs and absence of rod-shaped NPs in the current study. Therefore, the antimicrobial property of SPE based AgNPs may be influenced by method of production i.e., heating or photoinduced methods. The method of production is likely to change the properties of metallic NPs such as shape, size, charge, surface properties and presence of the phytochemicals etc^49,50^. Some previous studies reported that rod-shaped metallic NPs are lesser efficient than spherical NPs whereas smaller sized NPs possess higher cytotoxicity against microbial pathogens as compared to their larger sized NPs. Similarly, the charge on NPs defines their ability to bind with plasma membrane of mammalian cells. Therefore, the utility of metallic NPs for a specific application may be defined by the method of production^49–51^. Further, it has been reported that antimicrobial effect of AgNPs is likely due to the production of reactive oxygen species, enzyme inhibition and the penetration into cell via cell membrane etc^52,53^.

The de novo fabrication process for synthesizing silver nanoparticles with sunlight irradiation and using *S. persica* as reducing agent has potential applications in the fields of biomedical and environmental technologies e.g., the impregnation and immobilization of the NPs on various materials for wound treatment, water purification, and biosensing etc^54–56^. Moreover, other applications such as anticancer, anti-inflammatory, anti-coagulant, antioxidant etc.^6^ can also be explored using SL-SpNPs. Studying cytotoxicity of SL-SpNPs with human cell lines can also open new research directions towards different biomedical and therapeutic applications.

## Conclusion

Here, a de novo fabrication process is reported for synthesizing silver nanoparticles utilizing *S. persica* as reducing agent with sunlight irradiation. The reported fabrication process is simple, cost effective, eco-benign and fast (~ 10 min fabrication time) and resulted in generating a new class of less hazardous AgNPs which possess significant antibacterial activity. The characterization shows synthesized nanoparticles are spherical in shape with an average size of 15.38 nm, have phytochemical head groups and crystal structure. Further, SL-SpNPs show significant antibacterial efficacy against bacterial pathogens. MIC and MBC for *E. coli* were determined to be 1.5 μg/ml and 3.0 μg/ml respectively, while for *S. epidermidis* the values were 12.5 μg/ml and 25 μg/ml respectively. The current approach may be used directly or easily adapted to synthesize a wide range of other metallic nanoparticles as well. These eco-friendly AgNPs can be used for many biomedical applications.
